# Substantial and robust changes in microRNA transcriptome support postnatal development of the hypothalamus in rat

**DOI:** 10.1038/srep24896

**Published:** 2016-04-27

**Authors:** Soraya Doubi-Kadmiri, Charlotte Benoit, Xavier Benigni, Guillaume Beaumont, Claire-Marie Vacher, Mohammed Taouis, Anne Baroin-Tourancheau, Laurence Amar

**Affiliations:** 1 Université Paris-Saclay, Université Paris-Sud, CNRS, UMR 9197-Institut des Neurosciences Paris-Saclay, Orsay F-91405, France

## Abstract

MicroRNAs (miRNAs) modulate gene expression in male germ cells and somatic tissues of mammals on a genome-wide scale. Hundreds of miRNAs are encoded by mammalian genomes, a large fraction of which is expressed in brain. Here we have investigated the complexity and dynamics of miRNA transcriptomes that associate with neuronal network maturation of hypothalamic arcuate nucleus and median eminence (ARC/ME) in rat by analysing more than 300 miRNAs from 3–7 biological replicates at 5 postnatal time-points. The network connecting ARC/ME to other hypothalamic and extra-hypothalamic regions maturates in an environment-dependent manner. We therefore analyzed miRNA transcriptomes of progeny of dams fed either a balanced or unbalanced diet during gestation and lactation. More than 30% of the miRNAs displayed significative changes of expression between stages P8 and P14, and P21 and P28; half of the changes were greater than 3-fold. Among those miRNAs were well-known and dozens of still poorly documented miRNAs. Progeny of dams fed an unbanced diet displayed a severe growth retardation phenotype, lower levels of plasma leptin but almost identical miRNA transcriptomes. Together these data demonstrate that two substantial and robust changes in miRNA transcriptome of ARC/ME occur at a period crucial for neuronal network functional organization.

MicroRNAs (miRNAs) are RNA molecules of 20–24 bases that post-transcriptionally control the expression of protein-coding genes throughout interactions with complementary bases of target messenger RNAs (mRNAs). As a result, translation efficiency and/or stability of mRNAs are decreased and the levels of the corresponding proteins are diminished^1^,^2^.

Mammalian genomes harbor several hundreds of miRNA-encoding genes (miR genes) whose expression is essential during spermatogenesis and in somatic tissues of the post-implantation embryo and adult. Based on well-characterized interactions between miRNAs and target mRNAs involving base positions 2–8 (seed regions) in the former and complementary sequences within 3′UTRs of the latter, several algorithms have been developped for target prediction. Because of the short size of the predicted miRNA:mRNA heteroduplex and large amounts of sequences in mRNA transcriptomes, bioinformatics analyses predict that miRNAs modulate gene expression on a genome-wide scale, each miRNA controlling the expression of several hundreds of protein-coding genes. Bioinformatics analyses also predict that expression of most protein-coding genes are controlled by miRNA combinations. Therefore miRNA transcriptomes rather than single miRNA expressions should be investigated to provide full information on the control of gene expression.

Brain harbors complex miRNA transcriptomes, complete information of which can be recovered by high-throughput sequencing (HTS) technologies that provide digital data and unambiguous identification of miRNAs. miRNA transcriptome complexity (i.e. the multiplicity of miRNA sequences in miRNA transcriptomes) in brain comes in part from the large number of miR genes that are expressed^3^. Complexity of miRNA transcriptomes is also due to the fact that miRNAs do not maturate as single products of miR genes but as multiple products differing by one or a few bases at their ends (isomiRs). 3′-isomiRs (miRNAs with different 3′ ends) should target identical sets of mRNAs. Whether 3′-isomiRs display identical targeting efficiencies and/or half-life times is under investigation. In contrast, 5′-isomirs (miRNAs with different 5′ ends) that display different seed regions are expected to target different mRNA sets. Unambiguous identification of 3′- and 5′-isomiRs is therefore a challenge for miRNA research and research on the control of gene expression. Unambiguous identification of related miRNAs produced by miR multigene families is also a challenge. Such miRNAs that differ by one or a few bases at internal positions, downstream of the seed region, can target different mRNA sets^4^. Besides providing unambiguously identification of isomiRs and related miRNAs, HTS technologies have the advantage of producing hundreds of milllions of data at a (relatively) reasonable cost. This allows the study of high numbers of biological replicates and physiological conditions, thus the generation of data of statistical and biological significance.

The hypothalamic arcuate nucleus (ARC) harbors orexigenic Agouti-related peptide/Neuropeptide Y (AgRP/NPY) and anorexigenic pro-opiomelanocortin (POMC) neurons that communicate with second-order neurons located in a number of other hypothalamic as well as extra-hypothalamic regions to adjust food intake and energy expenditure to body needs. The ARC is located adjacent to the median eminence (ME) and 3^rd^ ventricle. While the former is one of the few brain regions devoid of blood brain barrier and as such, especially sensitive to circulating signals, the latter conveys circulating signals through cerebrospinal fluid. Those features make ARC/ME a primary center for the sensing of nutrient and hormone variations, integration of input signals and regulation of the energy balance.

The formation of the complex neuronal circuitry connecting the ARC to other hypothalamic and extra-hypothalamic regions includes axonal growth and synaptic maturation. This occurs during the first three weeks of life in rodents^5^ and developing ARCs of mice and rats in the early postnatal period are widely used models. Leptin plays a critical role in the formation of the ARC neuronal circuitry. Projections from the ARC are two to four times fewer in leptin-deficient *Lep*^*ob*^*/Lep*^*ob*^ adult mice relatively to *Lep*^+^*/Lep*^+^ adult mice^6^, and in adult rats of Diet Induced Obsesity strains compared to adult rats of Diet Resistant strains^7^. Projections from the ARC can be partly restored in *Lep*^*ob*^*/Lep*^*ob*^ adult mice by leptin administration during the maturation period^6^. Several studies have shown that the maternal diet and metabolic perinatal environment play critical roles in the formation of the ARC neuronal circuitry. Maternal high-fat diet during lactation impairs the ARC circuitry in mice^8^ while maternal perinatal undernutrition drastically reduces postnatal plasma leptin and affects the development of the ARC neuronal network in rats^9^. In mice as in rats, defects in the ARC neuronal circuitry correlate with Type 2 diabetes and/or obesity. Maternal unbalanced diet has also been shown to impact the organization of astrocytes in ARCs of young progeny^10^.

miRNA maturation depends upon the DICER endoribonuclease. Conditional deletions of *Dicer* in POMC-expressing cells result in a POMC neuron degenerative process that starts around 3 weeks of age, as well as defective glucose metabolism, obesity and alterations in the pituitary-adrenal axis in adult mice^11^,^12^,^13^. Degeneration of POMC-expressing cells establishes that miRNAs are key players for functional ARCs in neonates and adults.

Our work aimed to test two hypotheses. First, ARC/ME development and neuronal network maturation associate with substantial changes in miRNA populations. Second, these changes are largely influenced by the quality of the developmental environment. In studying more than 300 miRNAs in rat, we showed that the first hypothesis was true, while the second was not verified in our experimental conditions. Our data demonstrate that substantial and robust changes in miRNA transcriptome support the development of the hypothalamus during a period of high remodelling of the neuronal circuitry and provide a sound framework for further investigation of combined changes in miRNA and target mRNA expression.

## Results

### Complex miRNA transcriptomes characterize the hypothalamic ARC/ME between stages P4 and P28

In order to look for changes in miRNA transcriptome of ARC/ME in the period of axonal growth and synaptic maturation, we have first dissected the corresponding area of 6–7 male Wistar rat progeny of dams fed a balanced diet at postnatal stages P4, P8, P14, P21 or P28. Total RNAs have been extracted, treated with DNase or used to purify small RNAs of 18–36 bases. At each stage, progeny were obtained from three different litters in order to limit breeding and/or housing bias.

ARC harbors a neuronal population highly producing POMC transcripts. To characterize ARC/MEs, we determined the level of POMC transcripts relatively to that of ubiquitous GAPDH transcripts using DNA-free RNAs and RT-qPCRs (Supplemental Fig. S1A and corresponding legend). On the basis of their relative expression compared to that of hypothalamic paraventricular nuclei (PVN) taken as POMC-non-expressing controls, 3 ARC/MEs of stage P4 and 1 of stage P8 were discarded from our analysis. One ARC/ME of stage P28 which value was much lower than the other values of the same stage was also discarded. Four, 6, 6, 7 and 6 ARC/MEs were respectively conserved for further analysis of miRNA transcriptomes at stages P4, P8, P14, P21 and P28, respectively.

For each ARC/ME, an individual cDNA library was built using small RNAs and an Illumina-like protocol. Individual miRNA expression profiles were constructed from 1.4 +/− 0.3 millions of reads mapping to known miR genes (Supplemental Table S1A).

495 miR genes are listed for the rat genome in the miRBase database release 21, potentially encoding ∼2000 miRNAs (see Materials and Methods). Individual profiles of miRNA expression were used to build mean expression profiles in which more than 550 miRNAs were identified by more than 10 reads at, at least, one stage (Supplemental Table S2). Only those miRNAs were considered in our analyses. The choice of this limit was based on the fact that miRNAs of low expression in profiles built from tissues such as ARC/ME with several cell types can identify miRNAs of high abundance within one cell type. miRNAs of low expression in profiles can also identify miRNAs of moderate or high abundance in tissues as a consequence of a biased representation in cDNA libraries^14^. Note that biased representation is reproducible between cDNA libraries and has therefore no impact on analyses of miRNA differential expression.

### Differences of homogeneity in miRNA transcriptome across biological replicates suggest high dynamics of miRNA expression at stages P14 and P21

**Figure 1:**
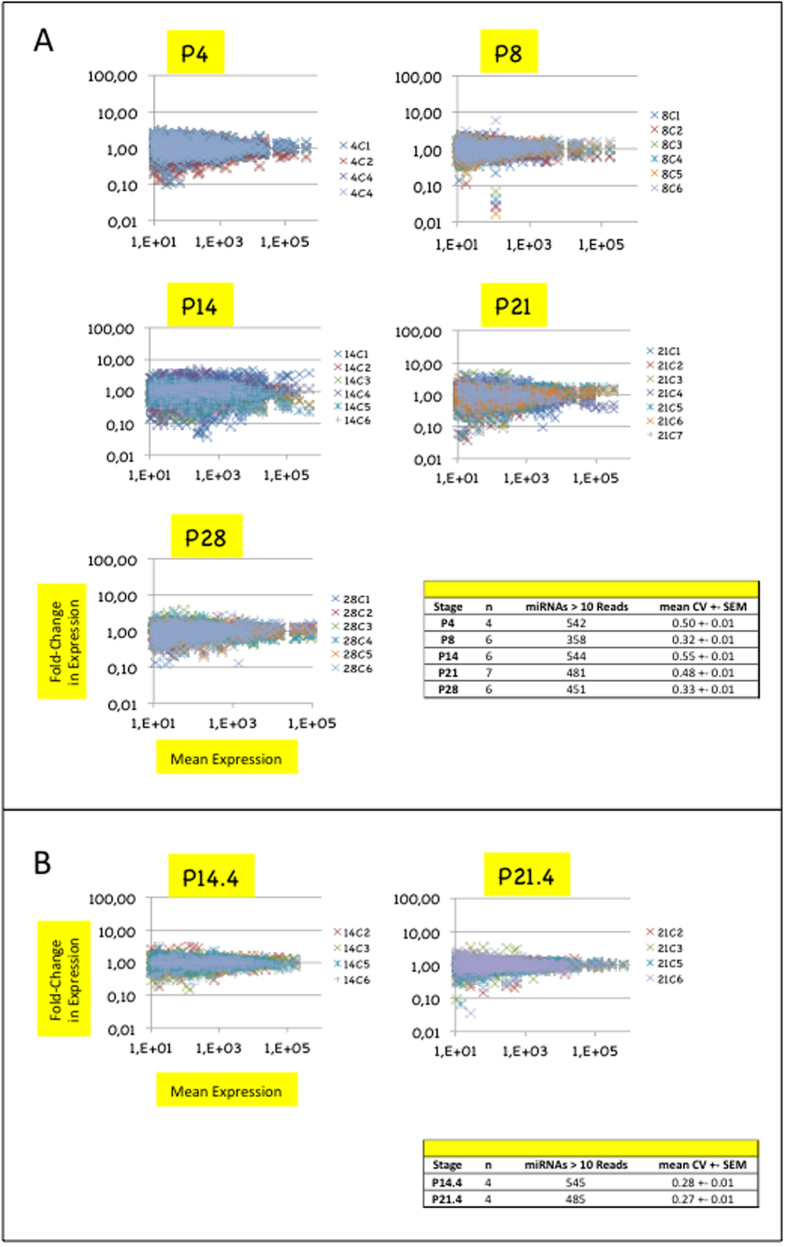
Differences in miRNA expression homogeneity across biological replicates suggest higher dynamics of miRNA expression in hypothalamic ARC/ME at stages P14 and P21. (**A**) Plots depict miRNA relative expressions of ARC/ME at P4, P8, P14, P21 or P28. Stages are indicated above each plot. In each plot, the X-axis denotes mean read counts between biological replicates, the Y-axis, fold changes of expression relatively to the mean expression. Only miRNAs identified by >10 reads are shown. X- and Y-axes are drawn using a log(10) scale. Each dot represents the relative expression of an individual miRNA expression and each color, a biological replicate. Dots of positive or negative values on the Y-axis denote up- or down-regulated miRNA expressions relatively to mean expressions, respectively. Values on the Y-axis close to 1 denote miRNAs of highly homogeneous expression. Note that many Y-values were distant from 1 for miRNAs of low mean expression. This accounted for the fact that the lower the mean expression, the less precise its quantification. Coefficients of variations (standard deviation/mean; CV) were calculated for each miRNA (see Supplemental Table S2), and global CV (mean +/SEM), for each stage. Table in bottom right corner summarizes data for each stage. (**B**) Sub-stages P14.4 and P21.4 could be identified at stages P14 and P21, respectively.

We first determined the level of homogeneity of miRNA expression at each stage by comparing miRNA expressions profiles of biological replicates to their corresponding mean profiles (Fig. 1A). Levels of homogeneity were quantified at each stage by computing the coefficients of variation of expression (ratios of the standard deviation to the mean) either over all miRNAs (global CVs) or over sliding windows of 50 miRNAs of decreasing abundance (sliding CVs) (see Fig. 1A and Supplemental Fig. S2A).

Stages P8 and P28 displayed similar global CVs (mean +− SEM = 0.32–0.33 +/− 0.01) and sliding CVs (ranging from 0.22 to 0.48). In contrast, stages P4, P14 and P21 displayed high global CVs (0.48–0.55 +/− 0.01) and sliding CVs. Stage P14 clearly enclosed a sub-stage P14.4 of four biological replicates which were characterized by much lower global CV (0.28 +/− 0.01) and sliding CVs ranging from 0.20 to 0.39 (Fig. 1B and Supplemental Fig. S2B). Stage P21 also clearly enclosed a sub-stage P21.4 of four biological replicates of low global CV (0.27 +/− 0.01) and sliding CVs (from 0.18 to 0.35). Sub-stages P14.4 and 21.4 were not litter-dependent.

Together, low homogeneity of miRNA expression across biological replicates at stages P14 and P21 and litter-independency of homogeneous expression at substages P14.4 and 21.4 suggested that a high dynamics of miRNA population takes place in the hypothalamic ARC/ME during a period of peaks of axon growth and synapse formation. Stages P14.4 and 21.4 were used in further analyses.

### miRNA transcriptomes display substantial changes between stages P8 and P28

**Figure 2:**
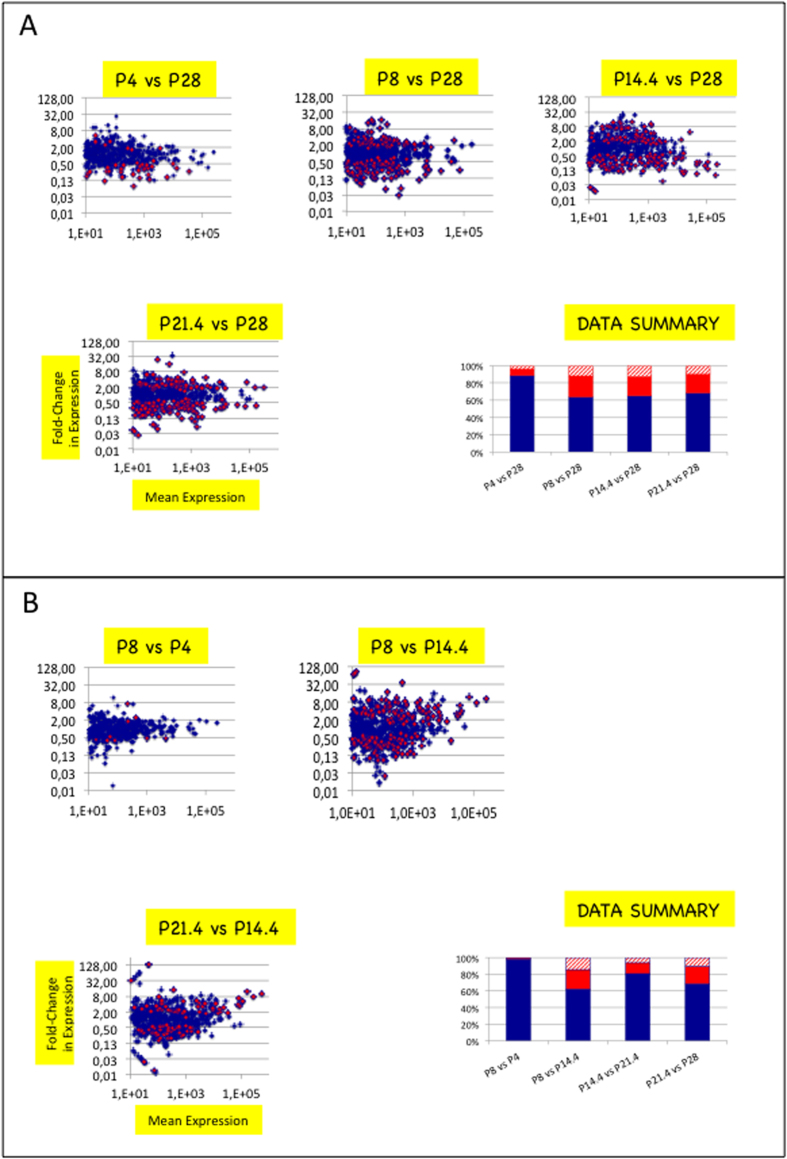
Substantial changes in miRNA expression mark ARC/ME development. (**A**) Scatter plots depict fold-change in miRNA expression of ARC/ME at P4, P8, P14.4 or P21.4 versus P28. In each plot, the X-axis denotes mean read counts between P4, P8, P14.4, P21.3 or P21.4 and P28, the Y-axis, fold changes in miRNA expression. Positive or negative values on the Y-axis denote up- or down-regulated miRNA expressions relatively to expression at P28, respectively. X- and Y-axes are drawn using log(10) or log(2) scales, respectively. Blue dots denote miRNAs with no significant expression change. Red dots denote miRNAs with expression changes of padj-values <0.05. p-values were calculated with the Mann and Whitney test and adjusted for multiple testing using the method described in Benjamini and Yekutieli^26^. In histogram in bottom right corner, blue bars identify percentages of miRNAs with no significant change, red bars identify percentages of miRNAs with significant changes >0.33 or <3 and hatched bars, miRNAs with significant changes <0.33 or >3 (sum = 100%). (**B**) Scatter plots depict fold-change in miRNA expression of ARC/ME between pairs of side-stages. miRNAs miR-298* and 532-3p in comparison P8 vs P14.4 and miR-193-3p in comparison P14.4 vs P21.4 are out of plots. For plots depicting changes occuring between P21.4 and P28, refer to (**A**).

We then compared miRNA expressions at stage P28 with miRNA expressions at stages P4, P8, P14.4 or P21.4 (Fig. 2A). p-values were corrected for false discovery rates and padj-values < 0.05 were retained as significant (see Materials and Methods). Depending on the comparison, 39–178 miRNAs (8–43%) exhibited expression changes and 22–99 of changes (5–22%) were higher than 3 (higher than 3.0 in cases of up-regulation and lower than 0.33 in cases of down-regulation).

**Figure 3:**
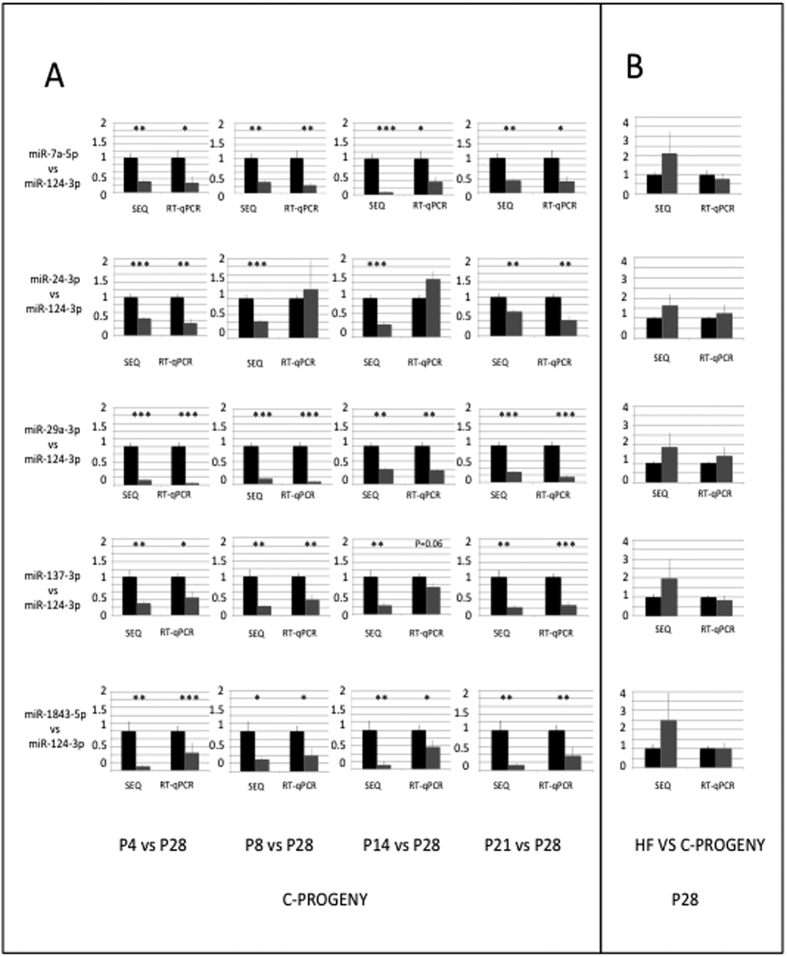
RTqPCR versus sequencing quantification of miRNA expression. (**A)** Ratios of miR-7a-5p, miR-24-3p, miR-29-3p, miR-137-3p and miR-1843-5p expression to miR-124-3p expression at P4, P8, P14.4 and P21.4 were compared to ratios at P28 using data recovered from HTS or RT-qPCR technologies. Data are organized in lines (one miRNA/line) and columns (one stage/column). The reference ratio of 1 at P28 is shown as black bars while ratios at P4, P8, P14.4, P21.3 and P21.4 are shown as grey bars. p-values < 0.05, 0.01 and 0.001 are identified by *^,^** and *** symbols, respectively. All data are expressed as mean +/− SEM. **(B)** Ratios of miR-7a-5p, miR-24-3p, miR-29-3p, miR-137-3p and miR-1843-5p expression to miR-124-3p expression were calculated at P28 for progeny born to dams fed a balanced diet and compared to those of progeny born to dams fed an unbalanced diet.

Our experimental scheme involved the sequencing of several replicates at each developmental stage or sub-stage to provide miRNA expression data of statistical significance. We also performed RT-qPCRs on the same RNA samples to validate sequencing data. Given the number of miRNAs and the limited quantity of material in individual ARC/MEs, we selected six miRNAs: miR-124a-3p which is widely expressed throughout brain^15^, miR-29a/b-3p whose brain-specific knockdown results in neuronal cell death^16^,^17^, miR-7a-5p and miR-137-3p which are expressed in hypothalamic nuclei including ARC^18^, miR-24-3p and miR-1843-5p which are still poorly or not clearly documented. As the U6 snRNA was excluded from small RNA fractions and could not been used as an internal reference to quantify miRNA expressions, we quantified the expression of miR-7a-5p, miR-24-3p, miR-29-3p, miR-137-3p and miR-1843-5p relatively to that of miR-124-3p, at P4, P8, P14.4 and P21.4 relatively to P28, from RT-qPCR amplification or sequencing data (Fig. 3A). Out of the 20 comparisons, RT-qPCR amplification and sequencing data gave similar information exept in the two cases of miR-24-3p at stage P8 and stage P14.

Together these data demonstrate that substantial changes in the miRNA transcriptome of ARC/ME occur during the first four postnatal weeks. The exhaustive list of miRNA expression comparisons is presented in Supplemental Table S3.

### miRNA transcriptomes of ARC/ME display at least two substantial changes between stages P8 and P28

To characterize the kinetics of changes in miRNA transcriptome of ARC/ME between stages P4 and P28, pairs of side-stages were compared (Fig. 2B). Stages P4 and P8 displayed different but similar miRNA transcriptomes. 9 miRNAs (2%) were differentially expressed; 1 miRNA (0.2%), miR-344g-hairp had expression differences higher than 3. In contrast, major changes support the transition between stages P8 and P14.4. In that case, 177 miRNAs (37%) were differentially expressed and 107 miRNAs (23%) had expression differences higher than 3. Changes in miRNA transcriptome also occured between stages P14.4 and P21.4. Ninety miRNAs (15%) were differentially expressed, 41 (8%) had expression differences higher than 3. Finally, widespread changes in miRNA transcriptome supported the transition between stages P21.4 and P28 : 151 miRNAs (32%) were differentially expressed among which 70 (15%) had expression differences higher than 3.

Altogether these results demonstrated that at least two substantial changes in miRNA transcriptome support ARC/ME maturation. In most cases, related miRNAs that are produced from multigenic families displayed coordinated regulations. The exhaustive list of miRNA expression comparisons between side-stages is presented in Supplemental Table S4.

### miRNA trancriptomes are highly similar in ARC/ME of progeny with impaired body growth and low plasma leptin

We also examined the impact of postanatal growth retardation on the miRNA transcriptome of developing ARC/ME by studying male Wistar progeny of dams fed an unbalanced maternal diet (16% of protein, 38% of carbohydrates and 46% of lipid-derived energy intake) during the gestation and lactation periods (HF-dams). Progeny from HF-dams (HF-progeny) were obtained at the same time than progeny of dams fed a standard balanced diet that we studied first (thereafter named C-progeny and C-dams, respectively). As for C-progeny, 6–7 male HF-progeny were obtained from three different litters at postnatal stages P4, P8, P14, P21 or P28.

**Figure 4:**
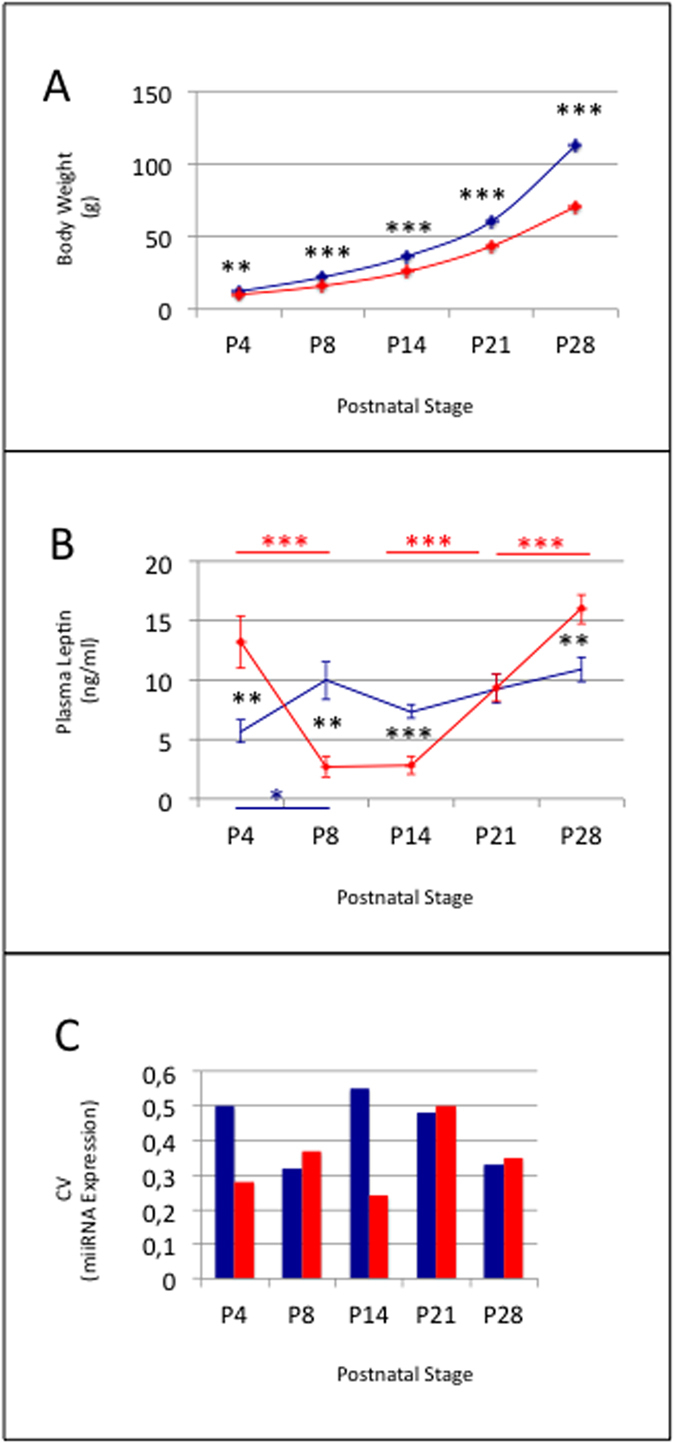
Maternal unbalanced diet impairs progeny body growth and plama leptin levels. Body weight **(A)**, plasma leptin levels **(B)** and global CVs of miRNA expression **(C)** in C- and HF-progeny. Data are shown as mean +− SEM in (**A**) and (**B**). C- and HF-progeny are identified by blue and red colors, respectively. Both progeny displayed high homogeneity in body weight at P4, P8, P14, P21 or P28, and hence very low SEMs. Symbols *^,^** and *** identified p-values <0.05, 0.01 and 0.001, respectively. Symbols in blue and red identify p-values calculated in side-stage comparisons in C- and HF-progeny, respectively. Black symbols identify p-values calculated in progeny comparisons.

From stage P4, HF-progeny displayed severe body growth retardation when compared to C- progeny (p < 0.01) (Fig. 4A). Growth retardation rose up to 30% at stage P28 (p < 0.001). HF-progeny also displayed severe variation of plasma leptin with a decrease by a factor of 2–3 at stages P8 and P14 and an increase by a factor of 1.5–2 at stages P4 and P28 when compared to C-progeny (p < 0.01 in all cases). The level of plasma leptin was similar between C- and HF-dams (Supplemental Fig. S3).

ARC/MEs were dissected. Total RNAs were extracted, treated with DNase, or used to purify small RNAs of 18–36 bases as above. Upon analyses of POMC transcripts relatively to that of GAPDH transcripts, 2 ARC/MEs of stage P4 and 3 of stage P14 were discarded from our analysis (Supplemental Fig. S1B). Three, 7, 4, 7 and 7 ARC/MEs were conserved for further analysis of miRNA transcriptomes at stages P4, P8, P14, P21 and P28, respectively.

**Figure 5:**
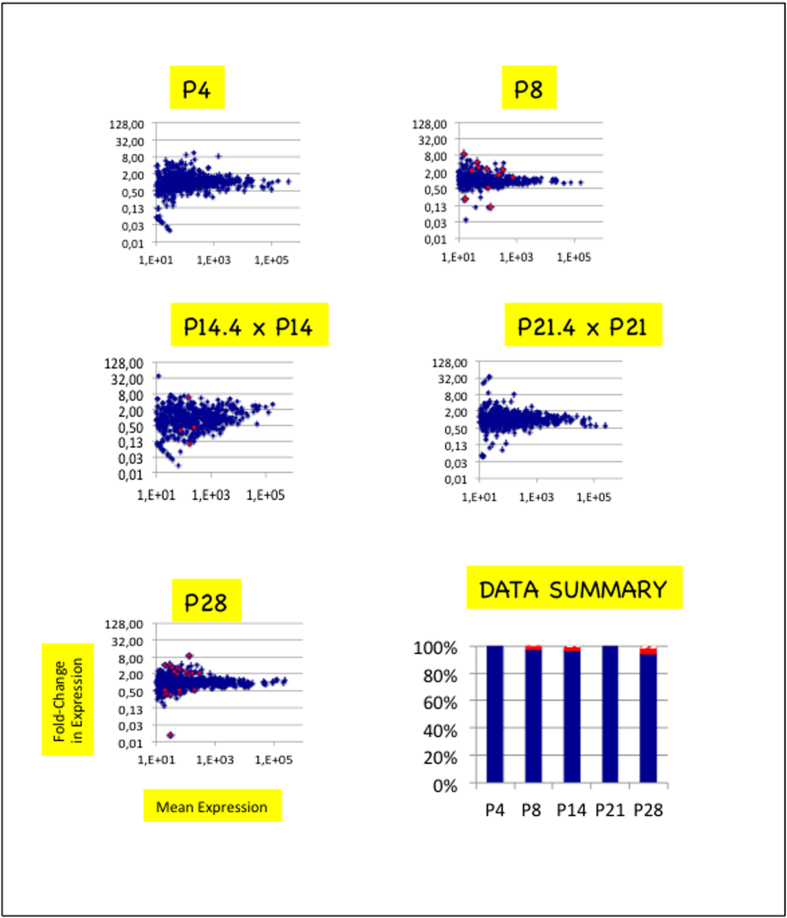
miRNA trancriptomes are almost unmodified in ARC/ME of HF-progeny. Scatter plots depict comparisons of miRNA expressions of ARC/MEs of progeny born to dams fed a balanced or unbalanced diet at P4, P8, P14, P21 or P28. See the legend of Fig. 2. In histogram in bottom right corner, only the blue and red bars can be seen. Hatched bars that account for <2% of the data are hardly seen.

cDNA libraries were constructed and miRNA expression profiles of ARC/MEs of HF-progeny were analyzed (Supplemental Figs S4 and S5, Supplemental Tables S1B & S5). miRNA expression homogeneities across biological replicates were similar in HF- and C- progeny at stages P8, P21 and P28 (Fig. 4C). Stages P4 and P14 were characterized by lower global CVs (0.24–0.25 +/− 0.01) and sliding CVs. miRNA expressions were then compared between C- and HF-progeny (Fig. 5, Supplemental Table S6). From P4 to P28, miRNAs displayed very similar expression between both progeny: 0–22 (<5%) miRNAs displayed differential expression and 0–7 of them (<2%) had expression differences higher than 3. Ratios of miR-7a-5p, miR-24-3p, miR-29-3p, miR-137-3p or miR-1843-5p expressions relatively to miR-124-3p expression calculated from RT-qPCR at P28 data matched those calculated from HTS data (Fig. 3B).

Despite large differences in postnatal growth and plasma leptin kinetics, miRNA transcriptomes were highly similar from P4 to P28 between C- and HF-progeny.

## Discussion

Our work aimed to test the hypotheses that neuronal network maturation in hypothalamic ARC/ME associates with substantial changes in miRNA transcriptomes, and if true, that these changes could be influenced by metabolic environments. miRNAs post-trancritionnally control gene expression in mammals. In tissues like brain that express a large fraction of miR genes, combination of miRNAs rather than single miRNAs are expected to participate in these controls. Combined miRNAs may act synergically or in opposition. As miRNAs associate with Argonaute 2 (AGO2) proteins to form and direct RNA-induced silencing complex (RISC) to target RNAs, miRNAs may also compete for AGO2 proteins if populations of the former overload cellular concentrations of the latter.

Here we demonstrate using HTS and RT-qPCR technologies, dissected ARC/MEs and large numbers of biological replicates, that substantial changes in miRNA expression occur during the period of functional organization of the ARC/ME neuronal network. More than 30% of the miRNAs display significative expression changes first between stages P8 and P14, then between stages P21 and P28. Half of the changes are greater than 3-fold. The power of miRNAs to regulate mRNAs is expected to be proportional to their abundance. All miRNA expression changes might therefore impact mRNA stability and/or translation efficiency.

To our knowledge, few studies have looked at miRNA population changes during the period of functional organization of the neuronal network of the hypothalamic ARC. Zhang *et al.*^19^ used a combined approach of NGS, microarray and RT-qPCR technologies to characterize the expression of 175 miRNAs in hypothalamus of pigs at P60, P120 and P180. Thirty-seven miRNAs, 13 of which are conserved among mammals, appeared differentially expressed between stages P60, P120 and/or P180. It is however unknown whether these stages encompass the period of neuronal network organization in ARC/ME. In consequence changes in miRNA expression profiles cannot be compared beween the rat and pig models.

More recently, Topper *et al.*^20^ used specific RT-qPCRs to characterize the expression of 8 miRNAs chosen for their abundance in hypothalamic profiles, importance in neurodevelopment, or sexual dimporphism, in the hypothalamic medial preoptic nucleus (MPN) and ventromedial nucleus (VMN) of male Wistar rats at postnatal stages P15, P30, P45 and P90. MPN is involved in osmoregulation, thermoregulation and in the control of sleep homeostasis while VMN participates in the control of energy homeostasis. Let-7b, miR-124a and miR-9 displayed no expression differences in MPN between P15 and P30 while let-7a, miR-7, miR-132, miR-145 and miR-219 displayed up-regulations. In contrast, all the eight miRNAs displayed up-regulations in VMN between the two stages.

Our data established that miR-124, miR-145 and miR-219 are steadily expressed in ARC/ME from stages P4 to P28 (see Supplemental Table S4). Our data also established the up-regulation of all members of the let-7 and miR-7 gene families, as well as that of the four miRNAs encoded by the miR-132/212 cluster, i.e. miR-132-3p, miR-132-5p, miR-212-3p and miR-212-5p, when comparing stages P14 and P28. miR-9 displayed a transient down-regulation at P21.

Althogether these studies strongly suggest that an up-regulation of most, if not all, members of the let-7 and miR-7 families and of the miR-132/212 cluster marks hypothalamus development while miR-9, miR-124a, miR-145 and miR-219 displayed nucleus-specific regulations of expression. By studying miRNA expression profiles at multiple and close time-points in the early postnatal period of rat, our study also demonstrate the expression of dozens of still poorly documented miRNAs, many of which are regulated at the time of ARC/ME development.

Maturation of ARC/ME neuronal network is controlled in part by leptin and maternal diet during the gestation and/or lactation periods. Maternal perinatal undernutrition in particular has been shown to drastically reduce postnatal leptin and affect the development of the ARC neuronal network in rats^9^. Here we have performed a comparison of miRNA transcriptomes in progeny of dams fed a balanced or unbalanced diet during gestation and lactation. HF-progeny started to display body growth retardation from P4 as well as a 2–3 fold decrease in plasma leptin at stages P8 and P14. Noteworthy, despite these large differences, miRNA transcriptomes appeared highly similar from stages P4 to P28 between C- and HF-progeny. Stage P28 displayed highest, also modest, differences with less than 5% of miRNAs being differentially expressed between both progeny and less than 2% showing expression differences higher than 3. Altogether these data show that retardation in postnatal growth and low plasma leptin levels do not associate with large differences in miRNA expression in ARC/ME during the period of neuronal network formation.

Together our data provide first demonstration for a robust dynamics of miRNA expression in maturating ARC/ME and highlight the importance of considering multiple miRNA expressions when analysing the regulation of gene expression during hypothalamic development and/or periods of neuronal network functional organization. Knowledge of miRNA transcriptome changes is important for deciphering gene expression regulation in maturating neuronal network and hypothalamic regions and our work provide a solid framework for further analyses of the impact of miRNA transcriptome changes on coding transcriptomes and proteomes.

## Methods

### Animals

All animals were maintained under a 12/12 Light/Dark cycle (lights on at 8 a.m.) with stable temperature (22 ± 1 °C). Virgin females Wistar of 4 weeks and males Wistar of 8 weeks from CER Janvier, France, have been habituated to our facilities for 4 weeks before breeding. Standard balanced diet (18%, 23% and 59% of the energy content derived from lipids, proteins and carbohydrates, respectively, Safe, France) and water were provided *ad libitum*. From day 1 of pregnancy and until weaning, dams were either maintained on the standard diet or shifted to an unbalanced diet (46%, 16% and 38% of the energy content derived from lipids, proteins and carbohydrates, respectively, Safe, France). At birth, litters were sized to ten pups to prevent lactational under- or overnutrition. At postnatal stages P4, P8, P14, P21 or P28, between 9 a.m. and 10 a.m, three dams and their progeny were anesthesied with isoflurane. Body weights were measured before killing by decapitation. Brains were removed, frozen in isopentate maintained at −40 °C and stored at −80 °C. Bloods were collected in the presence of 50 ul/ml of 160 U/ml heparin. All procedures were conducted according to the guidelines of laboratory animal care and were approved by the local governmental commission for animal research: Ethic Committee for animal experimentation of Paris Center and South # 59 (France), with authorization # 91-467.

### Leptin measurements

Leptin plasma levels were measured using Elisa according to the manufacturer’s protocol (Millipore). 25 ul of plasma were used for each point.

### ARC/ME dissection, RNA extraction and purification of short RNAs

The ARC/ME is ventrally adjacent to the 3rd ventricle. A 2–3 mm section was cut from progeny brains, 1 mm rostral to the optic chiasma and ARC/ME was punched with relation to the ventral part of the 3rd ventricle. ARC/ME-containing punches were named ARC/MEs. PVN is located dorsally and laterally to the 3rd ventricle. A 1 mm section was cut from adult brains immediately rostral to the optic chiasma and PVN was punched with relation to the dorsal part of the 3rd ventricle. PVN-containing punches, were named PVNs. ARC/MEs, and PVNs were stored individually in Ceramic Bead tubes (Ozyme) at −80 °C.

On purpose, ARC/ME- or PVN-contaning Ceramic Bead tubes were added QIAzol lysis reagent (Qiagen) (700 ul) and immediately homogeneized using a PreCellys homogenizer (PreCellys 24/Cryolys) for 20 s at room temperature. After addition of choloroform (150 ul), homogeneates were centrifugated 15 mn at 12,000 rpm to separate the organic and aqueous phases. The latter was recovered and nucleic acids, precipitated by adding one volume of isopropanol. Pellets were centrifuged, rinced twice with ethanol 70% and resuspended in H2O (20 ul). The yield of total RNA was around 1–2 ug.

Two-thirds of total RNAs were added an equal volume of formamide, heat at 70 °C for 3 min and loaded on on a denaturing urea (8M) polyacrylamide (17%) gel for size-fractionation. In all samples, the high quality of RNAs was checked by a lack of any smear and the fact that the tRNAs, 5S RNA and 5.8S RNA migrated as discrete bands. Small RNAs of 18–36 bases were eluted from the corresponding slices by overnight incubation in NaCl 0.4M (0.8 ml) at 4 °C under gentle shaking. Eluats were precipitated by adding 2.5 volumes of ethanol in the presence of glycogen (10 ng) and resuspended in H2O (10 ul).

One third of total RNAs were treated with 1 U of DNase RQ1 (Promega) in a volume of 50 ul for 30 min according in the provided buffer, then phenol extracted, precipitated with ethanol in presence of NaOAc 0.3M, rinced twice with ethanol 70% and resuspended in H2O (10 ul).

### cDNA library construction

Individual cDNA libraries were built following an Illumina-like protocol in which 3′- and 5′-adapters were sequentially ligated at the 3′ and 5′ ends of small RNAs, respectively, to allow for reverse transcription (RT) and amplification by polymerase chain reaction (PCR). Oligonucleotides are listed in Supplemental Table S7. 3′-Adaptor (25 pmoles) was adenylated as described^21^ using 1600 U of T4 DNA ligase (NEB) and the Quick Ligation reaction buffer (NEB) in a volume of 50 ul by overnight incubation at 37 °C.

The Illumina-like protocol that we used displayed some modifications compared to the protocol previously described^22^. A first mix was made of small RNAs (5 ul) and [0.25 uM] adenylated 3′-adapter (0.5 ul) in 0.2 ml Thermo-Tubes (Thermo Scientific). To prevent secondary structures, mix were heated for 3 min at 70 °C, then kept on ice while adding [50%] PEG 8000 (2.4 ul), 80 U of truncated T4 RNA ligase 2 K227Q (0.4 ul) and the corresponding buffer (1 ul) (all provided by NEB). Mix were incubated for 90 min at 25 °C. After addition of [1 uM] 3′-adapter (0.5 ul) and a new 70 °C/ice cycle, mix were added with 3 U of T4 RNA ligase 1 (0.4 ul) (NEB) and [10 mM] ATP (1 ul) and incubated for 90 min at 25 °C. In a third step, mix were added with [100 uM] RT-primer (0.5 ul), submitted to a 70 °C/ice cycle, then added with 60 U of the Superscript III reverse transcriptase (0.65 ul), [0.1 M] DTT (0.7 ul), and 5X buffer (3.1 ul) (all from Life Technologies) and [10 mM each] dNTP (0.7 ul), and incubated for 90 min at 50 °C. Finally, mix were added with [100 uM] 3′- PCR-primer (0.6 ul), [100 uM] 5′-PCR-primer (0.3 ul) and 2X Master Mix Phusion enzyme (NEB) (15 ul). Reaction mix were splitted into 2 tubes to enhance thermic exchange, denaturated for 1 min at 98 °C and submitted to 16 cycles of PCR (20 s at 98 °C, 30 s at 55 °C, 25 s at 72 °C). PCR products were size-fractionated on a 6% polyacrylamide gel so that ~100-bp cDNAs could be separated from the 75-bp primer dimers.

Early multiplexing strategies used tagged 3′-adaptors^21^. Although these adaptors only display 4–6 base differences out of 20 bases, they were later shown to introduce artefactual differences in miRNA expression^23^ (and A.B-T. and L.A. personal data). As a consequence, data relying on these multiplexing strategies seem to be reliable only for large (>5–10) expression differences. To avoid the introduction of artefactual differences, we developped a strategy that introduced barcodes at the PCR step. PCR works at temperatures that minimize differences of secondary structures between single-strand molecules. We used a set of 3′-PCR-primers with whole complementarity to the 3′-adaptor but a bulge of 2 bases at position 22. Control cDNA libraries were built from RNA of one ARC as described^20^. When necessary, efficiency factors were calculated and used to individually correct miRNAs. cDNA libraries built from biological replicates were barcoded with the same 3′-PCR-primer and sequenced in different lanes of an Illumina’s genome analyzer GAIIX.

### miRNA expression profiling

Sequencing quality was ascertained using the FASTQC program (http://www.bioinformatics.babraham.ac.uk/projects/fastqc). cDNA libraries were demultiplexed using our scripts on the basis of the first eleven bases of the 3′ adaptor and reads were trimmed from the 3′ adaptor sequence. Reads < 16 bases which could not be mapped on the genome were discarded. Reads >16 bases were grouped into unique sequences and analyzed with the miRanalyzer server (http://bioinfo5.ugr.es/miRanalyzer/miRanalyzer.php) to build individual miRNA expression profiles. This server used the miRBase database version 18 (http://www.mirbase.org) to identify miRNA sequences and additional databases to classify non-miRNA sequences. Sequences that did not fit these database categories but matched the rat reference genome, together with their flanking-sequences were tested for the encoding of putative novel miRNAs by using the RNAfold or miRDeep programs. No new miR gene could be identified. We allowed two mismatches with the BN/SsNHsd/Mcwi reference genome in order to take into account nucleotide differences that could reflect differences between the Wistar and reference genomes, or be introduced during the library construction, sequencing procedure, and/or nucleotide differences resulting from an editing process (only a few thousands of reads were missed when no mismatch was allowed for data alignment with the reference genome).

The denomination of miR-3p and miR-5p is currently replacing the denomination of miRNA and miRNA* that did not take into account the maturation of both strands. Note that denomination changes are in process in the miRBase database so that, depending on the miRNA, one or the other denomination were used. miRNA maturation also produce isomiRs (see Introduction). Throughout the text and Tables, 5′-isomiRs distinct from reference miRNAs in the miRBase database are identified as miR-X-hairp. 3′ isomiRs are grouped into one name.

miRNA expression profiles were normalized using the DESeq procedure^24^.

### RT-quantitative PCRs

POMC and GAPDH transcripts were analyzed by reverse transcribing 1 ul (30–60 ng) of DNA-free RNAs, using 20 U of the Superscript III reverse transcriptase, [0.1 M] DTT (0.3 ul), 5X buffer (1.2 ul) (all from Life Technologies), [10 mM each] dNTP (0.3 ul) and random primers in a volume of 6 ul. Mix were incubated for 90 min at 50 °C. RT products were ten-fold diluted. qPCRs were performed using 2.5 ul of the diluted RT-products, specific primers derived from the rat genes (IDs: 24664 and 24383) and 5 ul of SYBR oremix Ex Taq master Mix (Takara RR820A) in a volume of 10 ul using a Step-one apparatus. Samples were heated for 20 s at 95 °C and submitted to amplification cycles consisting of 2 s at 95 °C and 20 s at 60 °C. Levels of POMC transcripts were quantified relatively to those of GAPDH transcripts, and relatively to those of one progeny of stage P28, by using the ΔΔCt method^25^ and experimentally ascertained amplification efficiencies.

miRNA expression were analyzed by reverse transcribing 1 ul of diluted small RNAs (1 ul in 14 ul) into cDNAs using TaqMan^®^ MicroRNA Reverse Transcription Kit and specific TaqMan MicroRNA Assays listed in Supplemental Table S7 (Life Technology) in a volume of 6 ul. 2 ul of RT products were amplified using the TaqMan^®^ Universal PCR Master Mix (Life Technology) in a volume of 10 ul. Samples were heated for 20 s at 95 °C and submitted to amplification cycles consisting of 1 s at 95 °C and 20 s at 60 °C. Expression of miR-7a-5p, miR-24-3p, miR-29-3p, miR-137-3p and miR-1843-5p were quantified relatively to those of miR-124-3p, and relatively to those of stage P28, by using the ΔΔCt method^25^ and experimentally ascertained amplification efficiencies.

### Statistical measures

Individual group statistics were calculated using Mann and Whitney tests and corrected for multiple testing when necessary according to the false discovery rate method described by Benjamini and Yekurielli^26^. miRNA expression with adjusted p-values (padj-values) < 0.05 were considered as statistically significant. Data in the text and figures are given as mean +− SEM.

## Additional Information

**Accession codes:** Raw data files have been submitted at the SRA database (NCBI) under the study accession number SRP058705.

**How to cite this article**: Doubi-Kadmiri, S. *et al.* Substantial and robust changes in microRNA transcriptome support postnatal development of the hypothalamus in rat. *Sci. Rep.* **6**, 24896; doi: 10.1038/srep24896 (2016).

## Supplementary Material

Supplementary Information

Supplementary Dataset 1

Supplementary Dataset 2

Supplementary Dataset 3

Supplementary Dataset 4

Supplementary Dataset 5

Supplementary Dataset 6

Supplementary Dataset 7
