# Supplementary material for: Substantial and robust changes in microRNA transcriptome support postnatal development of the hypothalamus in rat
Source: Sci Rep. 2016 Apr 27;6:24896. doi: 10.1038/srep24896 (PMC4847009; doi:10.1038/srep24896)
Supplement: Supplementary Information [file srep24896-s1.pdf]

Substantial and robust changes in microRNA transcriptome support postnatal development of the hypothalamus in rat

Soraya Doubi-Kadmiri[1#], Charlotte Benoit[1,2#], Xavier Benigni[1,3], Guillaume Beaumont[1,4], Claire-Marie Vacher[1], Mohammed Taouis[1], Anne Baroin-Tourancheau[1] and Laurence Amar[1*]

[1] Université Paris-Saclay, Université Paris-Sud,
CNRS, UMR 9197-Institut des Neurosciences Paris-Saclay, Orsay F-91405, France
[2] Present address : Nodea Medical, 1 mail du Professeur Georges Mathé, Villejuif F-94800, France
[3] Present address : Institute of Plant Sciences - Paris-Saclay, 2 Rue Gaston Crémieux, Evry F-91000, France
[4] Present address : Laboratoire de Bioinformatique, Centre National de Génotypage, Commissariat à l'Énergie Atomique, 2 rue Gaston Crémieux, CP5721, Evry F-91057, France
[#] These authors contributed equally to this work

SUPPLEMENTARY INFORMATION

*** Corresponding author : laurence.amar@u-psud.fr**

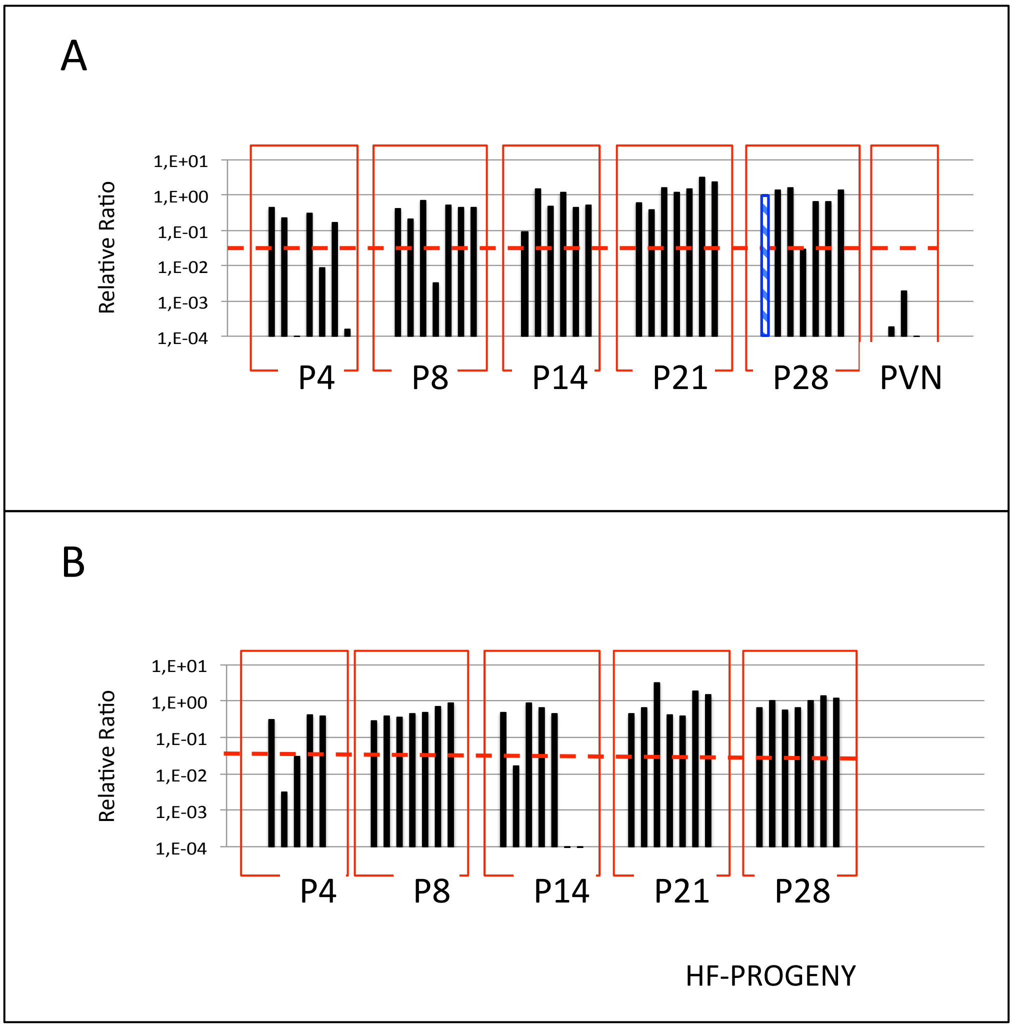

Figure S1. Assessment of ARC/MEs. (A) POMC transcripts were quantified relatively to GAPDH transcripts in ARC/MEs at stages P4, P8, P14, P21 or P28 by using RT-qPCRs. PVNs of 3 adults were used as POMC non-expressing controls. The relative ratio of POMC to GAPDH mRNAs of one ARC/ME at stage P28 (identified by a blue hatched bar) was taken as the reference ratio of 1. Note that the Y-axis is drawn using a log(10) scale. PVNs displayed relative ratios lower than 0.0019. The relative ratio of 0.019 (10 fold higher than the highest PVN relative ratio) was therefore taken as the threshold value for assessing ARC/ME. This threshold is drawn as a dashed red line. Three ARC/MEs of stage P4 and 1 of stage P8 were discarded. One ARC/ME of P28 which ratio was much lower than the other values of the P28 sample (lower than mean minus 1.7 standard deviation) was also discarded. **(B)** The same analysis was performed on ARC/MEs of HF-progeny at stages P4, P8, P14, P21 orP28. Two ARC/MEs of stage P4 and 3 of stage P14 were discarded.

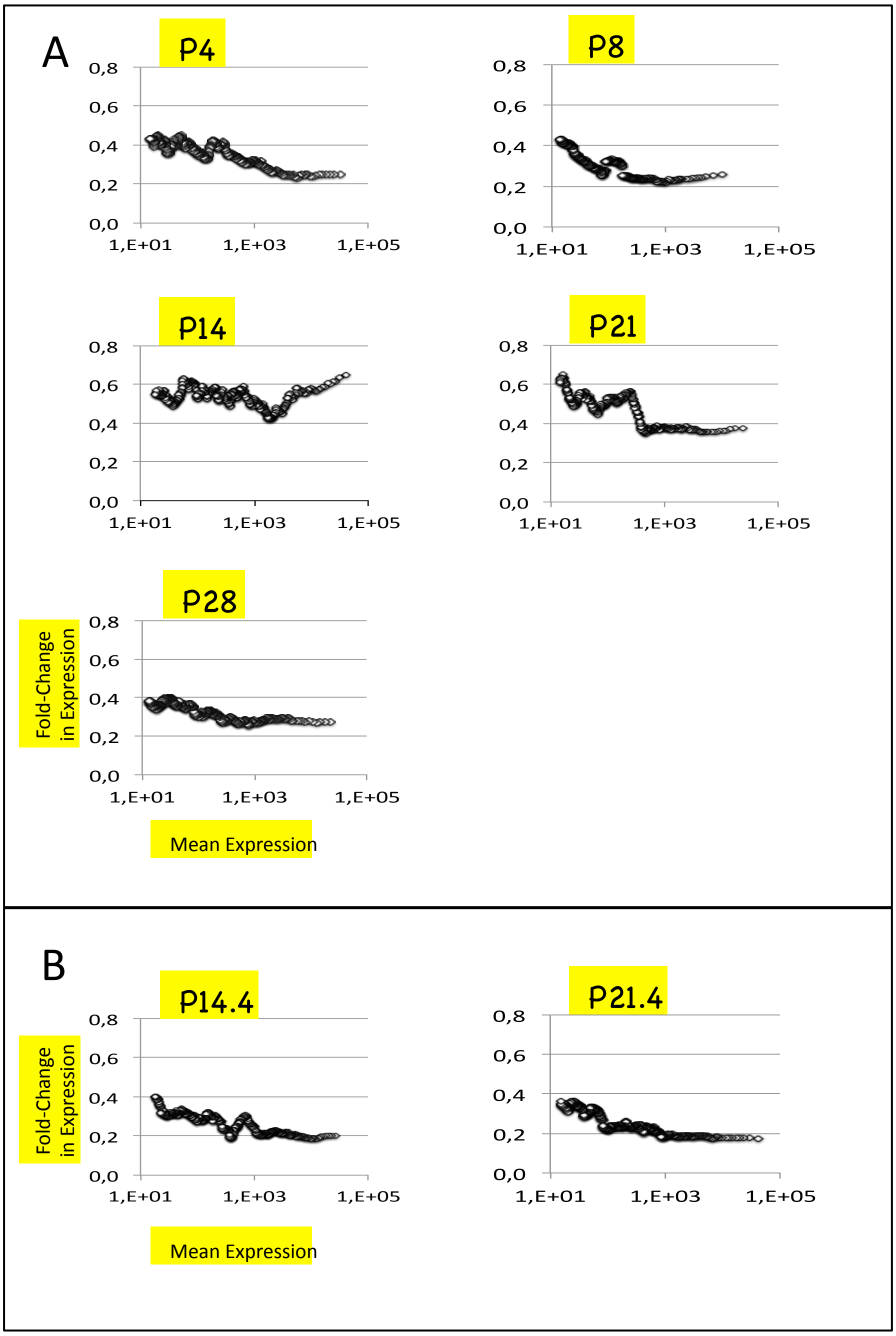

Figure S2. Quantification of miRNA expression homogeneity across biological replicates of ARC/ME. Levels of homogeneity were quantified by computing the coefficients of variation of expression (ratios of the standard deviation to the mean) either over all miRNAs (global CVs) (see Figure 1) or over sliding windows of 50 miRNAs of decreasing abundance (sliding CVs). Sliding CVs were plotted against mean expression of corresponding miRNAs. **(A)** Sliding CVs at stages P4, P8, P14, P21 or P28. Bulge at stage P8 is the consequence of the high CV of miR-328-3p (see Supplemental Table S2). **(B)** Sliding CVs at sub-stages P14.4 or P21.4.

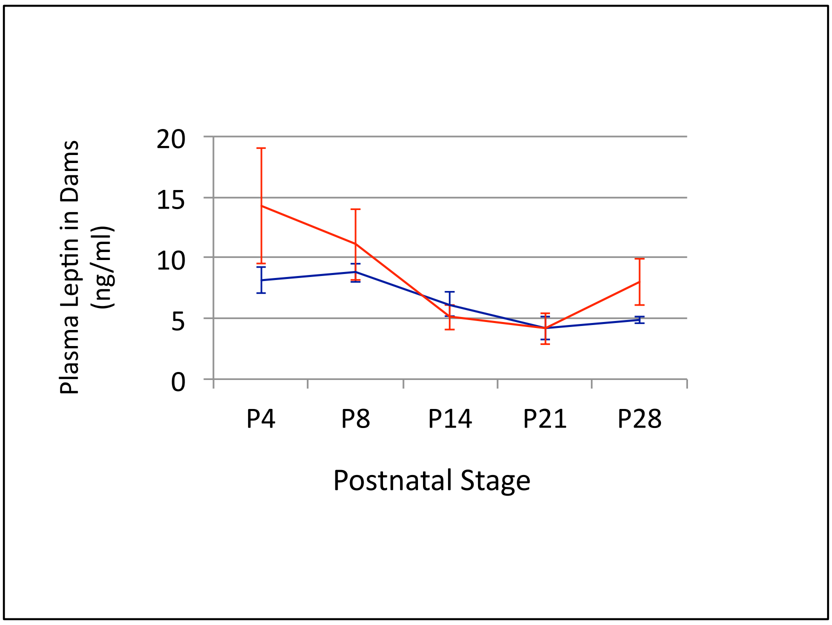

Figure S3. Dams of C- and HF-progeny display similar levels of plasma leptin during lactation. Data are shown as mean +- SEM. C- and HF-dams are identified by blue and red colors, respectively.

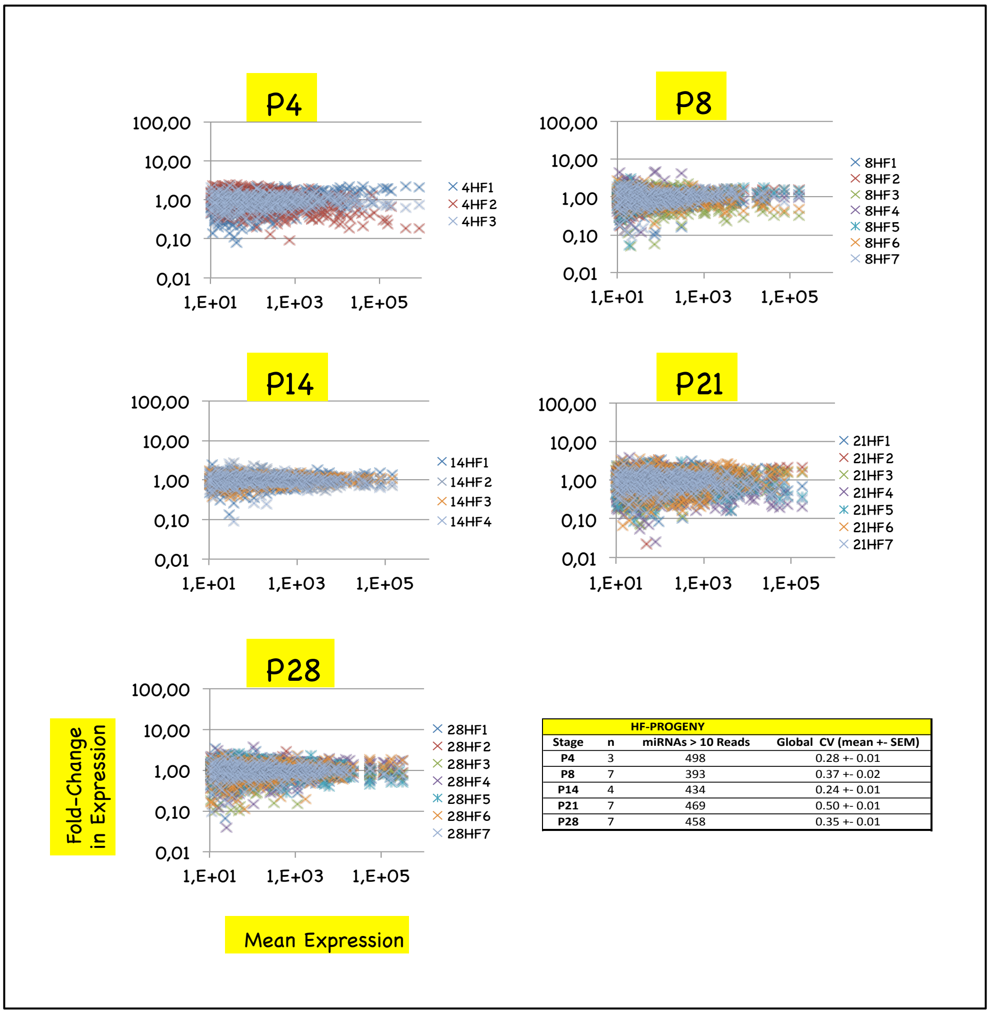

HF-PROGENY			
**Stage**	**n**	**miRNAs > 10 Reads**	**Global CV (mean +- SEM)**
**P4**	3	498	0.28 +- 0.01
**P8**	7	393	0.37 +- 0.02
**P14**	4	434	0.24 +- 0.01
**P21**	7	469	0.50 +- 0.01
**P28**	7	458	0.35 +- 0.01

Figure S4. Analysis of miRNA expression homogeneity across ARC/ME biological replicates in HF-progeny. Plots depict miRNA relative expressions of ARC/MEs at P4, P8, P14, P21 orP28. In each plot, the X-axis denotes mean read counts between biological replicates, the Y-axis, fold changes of expression relatively to the mean expression. X- and Y-axes are drawn using a log(10) scale. Each dot represents the relative expression of an individual miRNA expression and each color, a biological replicate. Dots of positive or negative values on the Y-axis denote up- or down-regulated miRNA expressions relatively to mean expressions, respectively. Values on the Y-axis close to 1 denote miRNAs of highly homogeneous expression. Many Y-values were distant from 1 for miRNAs of low mean expression. This accounted for the fact that the lower the mean expression, the less precise its quantification. Coefficients of variations (standard deviation/ mean; CV) were calculated for each miRNA (see Supplemental Table S5), and global CV (mean +/ SEM), for each stage. Table in bottom right corner summarizes data for each stage.

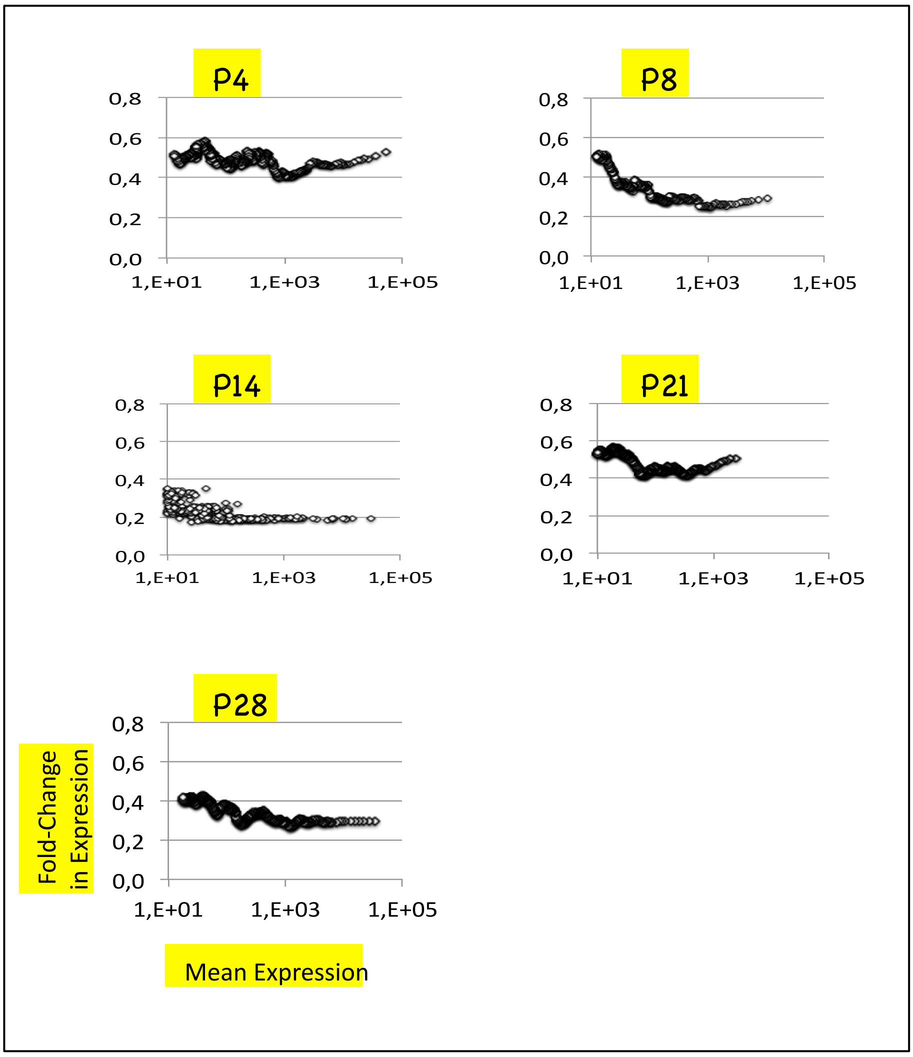

Figure S5. Quantification of miRNA expression homogeneity across biological replicates of ARC/ME in HF-progeny. Levels of homogeneity were quantified by computing the coefficients of variation of expression (ratios of the standard deviation to the mean) either over all miRNAs (global CVs) (see Supplemental Figure S4) or over sliding windows of 50 miRNAs of decreasing abundance (sliding CVs) at stages P4, P8, P14, P21 or P28. Sliding CVs were plotted against mean expression of corresponding miRNAs.

Supplemental Tables S1 – S7 are provided as individual .xls files**.**

Table S1: Sequencing data. Total read counts, counts and percentages of reads >16 bases and <40 bases, counts and percentages of miR-related reads among reads >16 bases and <40 bases are indicated for each cDNA library.

Tables S2-S6. In each Table, miRNAs are ranked by mean expression. Note that the denomination of miR-3p/miR-5p is currently replacing the denomination of miRNA/miRNA*. Denomination changes are in process so that one or the other denomination were used depending on the miRNA.

Table S2. miRNA expression profiles in developing ARC/MEs.

Table S3. Comparisons of miRNA expressions at P4, P8, P14.4, or P21.4 versus P28.

Table S4. Comparisons of miRNA expressions between side-stages.

Table S5. miRNA expression profiles in developing ARC/MEs of HF-progeny.

Table S6. Comparisons of miRNA expressions at P4, P8, P14 P21 and P28 between ARC/MEs of C- and HF-progeny. Note that comparisons at stages P14 and P21 involved C-progeny of sub-stages P14.4 and P21.4**.**

Table S7. Oligonucleotide Information. Oligonucleotides used for cDNA library construction and specific RT-qPCR analyses are indicated.
